# Physicians’ attitudes towards the development of the nurse prescribing role in critical care and emergency departments

**DOI:** 10.1186/s12912-023-01656-4

**Published:** 2023-12-19

**Authors:** Azam Naderi, Maryam Janatolmakan, Ziba Bolandi, Shahab Rezaeian, Alireza Khatony

**Affiliations:** 1 School of Nursing and Midwifery, Kermanshah University of Medical Sciences, Kermanshah, Iran; 2 Social Development and Health Promotion Research Centre, Health Institute, Kermanshah University of Medical Sciences, Kermanshah, Iran; 3 Infectious Diseases Research Centre, Health Institute, Kermanshah University of Medical Sciences, Kermanshah, Iran

**Keywords:** Physician, Critical care, Nurse prescribing, Nurse, Attitude

## Abstract

**Background:**

The progression of the nurse prescribing role encounters numerous challenges, with physician resistance being a significant obstacle. This study aims to assess physicians’ perspectives regarding the expansion of the nurse prescribing role within critical care and emergency departments.

**Methods:**

This cross-sectional study employed convenience sampling to enroll 193 physicians. Data collection instruments included a demographic information form and a researcher-developed questionnaire. Descriptive and inferential statistics were used to analyze the data using SPSS-22 software.

**Results:**

A total of 193 physicians participated in the survey, with a mean age of 41.9 ± 10.7 years. Among physicians from various age groups, genders, educational backgrounds, and clinical experiences, more than 60% acknowledged prescribing medicine as an essential component of their professional responsibilities. However, a significant majority of physicians in these categories agreed that in emergency situations, nurses should be allowed to prescribe medication to save patients’ lives. It is worth noting that, unlike specialist and fellowship physicians, a majority of general practitioners (83.3%) held the view that nurse-prescribed medications do not contribute to the professional development of nursing. The nurse prescribing role encountered several predominant obstacles, namely legal consequences (78.8%), interference of duties between physicians and nurses (74.1%), and a legal vacuum (77.2%).

**Conclusion:**

The majority of physicians expressed a favorable attitude towards nurse prescribing in emergency and critical care departments. To facilitate the development of the nurse prescribing role, it is essential to ensure the acquisition of scientific qualifications and implement necessary changes in nursing curricula across bachelor’s, master’s, and doctoral programs.

## Introduction

The nursing profession is witnessing the emergence of new competencies in various regions of the world [1]. Over the past two decades, one such responsibility that has gained prominence within the nursing role is prescribing medication [2]. Notably, the number of countries granting nurses the authority to prescribe medications has significantly risen in recent years [3]. Nurse prescribing offers several advantages, including enhanced patient care quality, improved continuity of care, time-saving for physicians, and cost-effectiveness [4–6]. In critical care units, the legalization of nurse prescribing holds particular significance [7]. These units face notable challenges, including the absence of physicians at all times and the need for nurses to obtain physician signatures for drug prescriptions [8]. Evidence indicates that many countries permit intensive care nurses to legally prescribe medication [9]. However, prescribing within the intensive care unit requires precision and sensitivity and should only be undertaken by experienced nurses [10]. Obtaining legal authorization to prescribe medication poses one of the most demanding requirements for nurses worldwide, with each country following a distinct process [11]. Despite nurse prescribing not being legally recognized in Iran, informal practices have been observed in various departments, particularly in emergency and critical care settings [10].

The advancement of the nurse prescribing role encounters various obstacles, with physician resistance being the most prominent barrier [12, 13]. Negative attitudes among physicians significantly impede the progression of this role [14]. Studies investigating physicians’ attitudes towards the development of the nurse prescribing role have yielded diverse findings. Some studies demonstrate physician agreement with role expansion, highlighting numerous benefits such as time-saving and reduced physician workload [15], improved patient access to medication, and enhanced communication between physicians and prescribing nurses [16]. Conversely, other studies have reported physicians expressing opposition to nurse prescribing [17, 18]. Reasons cited for their opposition include nurses’ insufficient preparation, limited knowledge of medical treatments, and uncertainty regarding the purpose of the nurse prescribing role [12, 19]. Notably, the primary challenge in developing the nurse prescribing role lies with physicians in critical care and emergency departments [13]. To our knowledge, no prior investigations have explored Iranian physicians’ attitudes towards the development of the nurse-prescribing role in critical care and emergency departments. Therefore, this study aims to assess physicians’ attitudes in this context, offering insights for role development and serving as a foundation for future research. The research questions for this study were as follows:

1. What are the attitudes of physicians towards the development of the prescribing role by nurses in critical care and emergency departments?
2. What are the attitudes of physicians towards the obstacles faced by nurses in prescribing medication in critical care and emergency departments?

## Methods

### Study design

This cross-sectional study was conducted between May 5, 2021, and September 22, 2022, within the critical care and emergency departments of hospitals affiliated with Kermanshah University of Medical Sciences. The findings were reported following the STROBE criteria (19).

### Sample and sampling method

The target population consisted of all physicians employed in the critical care and emergency departments of seven hospitals affiliated with Kermanshah University of Medical Sciences, Iran. The total number of physicians in this population was 257. Based on the sample size formula for a finite population, a sample size of 154 physicians was estimated, considering a confidence level of 95% and a margin of error of 5%. To account for potential non-respondents, an additional 25% was added to the sample size, resulting in a total of 193 physicians recruited for the study. The inclusion criteria were willingness to participate in the study and a minimum of one year of experience working in either critical care or emergency departments. Convenience sampling was employed for participant selection.

### Instruments

The data collection instruments consisted of a demographic information form and a researcher-developed questionnaire comprising three parts. The demographic information form consisted of eight items pertaining to age, gender, marital status, education level, expertise, clinical work experience, work experience in critical care or emergency departments, and workplace. The first part of the questionnaire, comprising 17 items, assessed physicians’ attitudes towards the necessity of nurse prescribing in critical care and emergency departments. Sample questions from this section include: “Prescribing medicine is solely the responsibility of physicians,” “Nurses do not have the legal authority to prescribe medication,” and “Nurses do not receive adequate preparation for prescribing during their education.” The second part of the questionnaire included five items investigating physicians’ attitudes towards nurse prescribing in critical care and emergency departments. These questions were of a yes/no type. Examples of questions in this section are: “The nurses in critical care and emergency departments possess the necessary competencies to prescribe medication,” “It is essential to develop the nurse prescribing role in critical care and emergency departments,” and “If the nurses in critical care and emergency departments undergo training courses and obtain legal authorization, I agree with their prescription of medication.”

The third part of the questionnaire consisted of 10 items focusing on the barriers to nurse prescribing in critical care and emergency departments. These items were rated on a five-point Likert scale ranging from “completely disagree” (1) to “completely agree” (5). Examples of items in this section include “Legal vacuum,” “Lack of clinical experience regarding diseases,” and “Lack of sufficient knowledge about disease management.” Participants were asked to indicate their level of agreement or disagreement with each statement.

The validity of the questionnaire was assessed using the qualitative content validity method. The questions were evaluated for relevance, simplicity, and clarity. To ensure the questionnaire’s validity, it was reviewed by seven nursing faculty members and five faculty members from various medical fields, including anesthesia and critical care, internal medicine, cardiology, and nephrology. Their feedback and suggestions were incorporated into the questionnaire. The reliability of the questionnaire was examined using the internal consistency method. To do this, the questionnaire was administered to a group of 15 physicians who were not part of the actual study. The internal consistency of the questionnaire was measured by calculating the alpha coefficients for each part. The obtained alpha coefficients for the first, second, and third parts of the questionnaire were 0.77, 0.78, and 0.79, respectively. These coefficients indicate a satisfactory level of internal consistency for the questionnaire.

### Data collection

Once ethical approval was obtained, the third author of the study visited the critical care and emergency departments to conduct data collection. During these visits, the objectives of the study were discussed with the physicians working in these departments. Those who expressed their willingness to participate were given the questionnaire. The questionnaire was administered to the participating physicians, and they were given approximately 30 min to complete it.

### Statistical analysis

The collected data were analyzed using the 22nd version of the Statistical Package for Social Sciences (SPSS v.22; SPSS Inc., Chicago, IL, USA) for data management and analysis across various disciplines SPSS was originally developed by Norman H. Nye et al. (1968) at Stanford University [20]. This study employed both descriptive and inferential tests to analyze and manage the data. Specifically, the Chi-square test or Fisher’s exact test was used for inferential analysis to investigate the differences in physicians’ attitudes based on demographic variables. Descriptive summaries were provided for qualitative variables such as demographic characteristics and physicians’ attitudes, through the calculation of frequencies and percentages. Mean and standard deviation were used to describe quantitative variables. The total number of physicians with positive or negative attitudes towards nurse prescribing was determined by combining the responses of “completely agree” and “agree,” as well as merging the responses of “completely disagree” and “disagree.” The denominator of the fraction included all the responses falling into these four categories.

To determine the number of physicians with an agreeing attitude, the sum of “completely agree” and “agree” responses was divided by the total number of responses in the four categories of “completely disagree,” “disagree,” “completely agree,” and “agree.” Conversely, to calculate the number of physicians with an opposing attitude, the sum of “completely disagree” and “disagree” responses was divided by the total number of responses in the four categories of “completely disagree,” “disagree,” “completely agree,” and “agree.”

### Ethical considerations

The study received ethical approval from the Ethics Committee of Kermanshah University of Medical Sciences with the code IR.KUMS.REC.1400.573. Prior to participation, all participants were provided with clear explanations regarding the study’s objectives. Written informed consent was obtained from each participant, indicating their voluntary agreement to participate in the study. Participants were assured that their personal details and information would be kept confidential and handled with the utmost care. The study followed ethical guidelines, including those outlined by national, international, institutional regulations, and the principles stated in the Declaration of Helsinki for research involving human subjects.

The study included a total of 193 physicians who completed the questionnaire, resulting in a response rate of 100%.

## Results

The participants had a mean age of 41.9 ± 10.7 years. The majority of the physicians, 62.2% (n = 120), fell within the age range of 31–50 years. The average working experience of the participants was 13.0 ± 9.0 years. Regarding the workplace distribution, 35.8% of the physicians (n = 69) worked in the general intensive care unit (Table 1).

**Table 1:** Demographic characteristics of physicians based on agreement with nurse prescribing role

Variables		Total	Agreeing with nurse prescribing role, n(%)
Age (y)	< 30	31(16.1)	12(12.8)
	31–50	120(62.2)	65(69.1)
	> 51	42(21.7)	17(18.1)
Gender	Male	112(58.1)	53(56.4)
	Female	81(42.0)	41(43.6)
Marital status	Single	79(40.9)	41(43.6)
	Married	114(59.1)	53(56.4)
Clinical work history, (y)	< 10	91(47.2)	48(51.1)
	11–20	69(35.7)	34(36.2)
	> 21	33(17.1)	12(12.7)
Work experience in critical care or emergency departments, (y)	< 10	96(49.7)	50(53.2)
	11–20	72(37.3)	36(38.3)
	> 21	25(13.0)	8(8.5)
Degree	General Physician	6(3.1)	0(0.0)
	Specialist	155(80.3)	80(85.1)
	Fellowship	32(16.6)	14(14.9)
Workplace	NICU^a^	28(14.5)	10(10.6)
	HICU^b^	37(19.2)	23(24.5)
	PICU^c^	40(20.7)	12(12.8)
	GICU^d^	69(35.8)	38(40.4)
	SICU^e^	19(9.8)	11(11.7)

^a^ Neonatal Intensive Care Unit; ^b^ High-Intensity Care Unit; ^c^ Pediatric Intensive Care Unit; ^d^ General Intensive Care Unit; ^e^ Surgical Intensive Care Unit

More than 70% of physicians across various age groups, genders, educational backgrounds, and clinical experience levels expressed a favorable opinion towards nurse prescribing in emergency situations, considering it necessary for saving patients’ lives. However, this finding was not statistically significant. Additionally, over 60% of physicians in different age groups, genders, educational backgrounds, and clinical experience levels recognized prescribing medications as part of a physician’s responsibilities. However, there was no statistically significant difference among physicians in these categories.

Among all physicians under the age of 30, 58.1% (n = 18), and among general practitioners, 83.3% (n = 5), were opposed to nurse prescribing in specialized departments and emergency even after completing training courses and obtaining legal authorization, and this finding was statistically significant (*P* = 0.025 and *P* = 0.030, respectively). Furthermore, 54.8% (n = 17) of physicians under the age of 30 and 83.3% (n = 5) of general practitioners disagreed with the notion that nurse prescribing contributes to the professional growth of nursing, and this finding was also statistically significant (*P* = 0.001 and *P* = 0.019, respectively).

The results showed that more than 40% of physicians in all three categories of clinical experience agreed that nurse prescribing contributes to the professional growth of nursing, and this finding was statistically significant (*P* = 0.004). However, 41.9% (n = 13) of physicians under the age of 30 disagreed with the notion that nurse prescribing improves the quality of patient care, and this finding was statistically significant (*P* = 0.034). In other age groups, genders, educational backgrounds, and clinical experience levels, more than 50% of physicians agreed with this perspective.

Among general practitioners, 66.7% (n = 4) were opposed to the necessity of expanding the role of nurse prescribing in specialized and emergency care departments, but this finding was not statistically significant. However, in other categorizations based on age, gender, educational background, and clinical experience, more than 50% of physicians agreed with the need for expanding the role of nurse prescribing in specialized and emergency care departments.

The majority of physicians in various age, gender, education, and clinical experience categories expressed that nurses do not have the legal authority to prescribe medication, and statistically significant differences were not found. Nineteen physicians in the age group under 30 (61.3%) and all general practitioners disagreed with the statement: “Nurses in specialized and emergency care units have the necessary qualifications to prescribe medication.” This finding was only significant in the education classification (*P* = 0.020), and in other categorizations, physicians agreed with this issue. Furthermore, 71.0% (n = 22) of physicians in the age group under 30 and 83.3% (n = 5) of general practitioners stated their disagreement with the statement: “Currently, there is the possibility of nurses prescribing medication in specialized and emergency care units.” This finding was statistically significant in the age groups (*P* = 0.006). Additionally, 61.3% (n = 19) of physicians under 30, 59.5% (n = 19) of physicians over 51 years old, 52.7% (n = 59) of all physicians with general education, 56.3% (n = 18) of physicians with fellowship specialization, and 54.9% (n = 56) of physicians with more than 10 years of clinical experience were opposed to nurses prescribing medication in specialized and emergency care units. This finding was only statistically significant in the education classification (*P* = 0.036).

More than 70% of physicians in various age, gender, and clinical experience categories agreed to the prescription of a limited number of emergency drugs by nurses after consulting with a physician. This is in contrast to all general practitioners who were opposed to this issue, but statistically, this finding was not significant in the education classification. Over half of the physicians in different age, gender, education, and clinical experience categories agreed with the following statements: “Nurse prescribing limits the scope of physician authority,” “Nurse prescribing harms the position of physicians in relation to patients,” “Nurses lack the necessary knowledge for prescribing,” “Nurses are not adequately prepared for prescribing during their education,” “Nurse prescribing does not align with the nature of the nursing profession,” and “The authority to prescribe medication should only be granted to nurses with a master’s degree or higher.” Regarding the statement “Nurses are not adequately prepared for prescribing during their education,” a statistically significant difference was found among physicians in terms of age (*P* = 0.006) (Table 2).

**Table 2:** Physicians’ attitudes towards the role of prescribing nurses based on demographic characteristics (N = 193)

Statement, n(%)	Responses^*^	Variables							
		Age, in year			Degree			Clinical work history, in year	
		< 30	31–50	> 51	General Practitioner	Specialist	Fellowship	≤ 10	> 10
In emergency situations, the nurse must be authorized to prescribe medicine.	Agree & totally agree	23(74.2)	91(75.8)	30(71.4)	5(83.3)	114(73.5)	25(78.1)	72(79.1)	72(70.6)
	Disagree & totally disagree	3(9.7)	15(12.5)	5(11.9)	0(0.0)	19(12.3)	4(12.5)	9(9.9)	14(13.7)
	P-value^**^	0.937			1.00			0.333	
Nurse prescribing saves the lives of patients in emergency situations.	Agree & totally agree	19(61.3)	91(75.8)	29(69)	4(66.7)	113(72.9)	22(68.8)	65(71.4)	74(72.5)
	Disagree & totally disagree	4(12.9)	7(5.8)	4(9.5)	0(0.0)	11(7.1)	4(12.5)	7(7.7)	8(7.8)
	P-value	0.269			0.535			0.994	
Prescribing medicine is only within the scope of the physician’s duties.	Agree & totally agree	23(74.2)	76(63.3)	30(71.4)	5(83.3)	100(64.5)	24(75.0)	56(61.5)	73(71.6)
	Disagree & totally disagree	4(12.9)	24(20.0)	4(9.5)	0(0.0)	30(19.4)	2(6.3)	20(22.0)	12(11.8)
	P-value	0.275			0.148			0.053	
If the nurses of critical care and emergency departments participate in training courses and obtain a legal license, I agree with prescribing medicine by them.	Agree & totally agree	13(41.9)	80(66.7)	22(52.4)	1(16.7)	98(63.2)	16(50.0)	59(64.8)	56(54.9)
	Disagree & totally disagree	18(58.1)	40(33.3)	20(47.6)	5(83.3)	57(36.8)	16(50.0)	32(35.2)	46(45.1)
	P-value	0.025			0.030			0.160	
Nurse prescribing helps the professional development of nursing.	Agree & totally agree	6(19.4)	83(69.2)	25(59.5)	1(16.7)	94(60.6)	19(59.4)	46(50.5)	68(66.7)
	Disagree & totally disagree	17(54.8)	22(18.3)	9(21.4)	5(83.3)	37(23.9)	6(18.8)	28(30.8)	20(19.6)
	P-value	0.001			0.019			0.036	
Nurse prescribing can threaten patients’ lives.	Agree & totally agree	19(61.3)	63(52.5)	18(42.9)	3(50.0)	80(51.6)	17(53.1)	45(49.5)	55(53.9)
	Disagree & totally disagree	4(12.9)	42(35.0)	13(31.0)	1(16.7)	47(30.3)	11(34.4)	28(30.8)	31(30.4)
	P-value	0.099			0.937			0.764	
Prescribing is not in the nurses’ job description.	Agree & totally agree	22(71.0)	71(59.2)	23(54.8)	5(83.3)	93(60.0)	18(56.3)	54(59.3)	62(60.8)
	Disagree & totally disagree	3(9.7)	34(28.3)	13(31.0)	1(16.7)	40(25.8)	9(28.1)	22(24.2)	28(27.5)
	P-value	0.077			0.777			0.762	
Nurse prescribing increases nursing professional independence.	Agree & totally agree,	6(19.4)	77(64.2)	27(64.3)	1(16.7)	88(56.8)	21(65.6)	43(47.3)	67(65.7)
	Disagree & totally disagree	15(48.4)	20(16.7)	8(19.0)	4(66.7)	35(22.6)	4(12.5)	28(30.8)	15(14.7)
	P-value	0.001			0.016			0.004	
Nurse prescribing improves the quality of patient care.	Agree & totally agree	11(35.5)	72(60.0)	25(59.5)	3(50.0)	88(56.8)	17(53.1)	46(50.5)	62(60.8)
	Disagree & totally disagree	13(41.9)	25(20.8)	11(26.2)	2(33.3)	40(25.8)	7(21.9)	25(27.5)	24(23.5)
	P-value	0.034			0.871			0.326	
It is necessary to develop nurse prescribing role in critical care and emergency departments.	Agree & totally agree	16(51.6)	66(55.0)	25(59.5)	2(33.3)	88(56.8)	17(53.1)	53(58.2)	54(52.9)
	Disagree & totally disagree	15(48.4)	54(45.0)	17(40.5)	4(66.7)	67(43.2)	15(46.9)	38(41.8)	48(47.1)
	P-value	0.817			0.525			0.460	
Nurses are not legally competent to prescribe medicine.	Agree & totally agree	19(61.3)	61(50.8)	27(64.3)	5(83.3)	84(54.2)	18(56.3)	45(49.5)	62(60.8)
	Disagree & totally disagree	5(16.1)	45(37.5)	10(23.8)	1(16.7)	48(31.0)	11(34.4)	31(34.1)	29(28.4)
	P-value	0.066			0.718			0.231	
The nurses of critical care and emergency departments have the necessary competence to prescribe medicine.	Agree & totally agree	12(38.7)	73(60.8)	21(50.0)	0(0.0)	88(56.8)	18(56.3)	51(56.0)	55(53.9)
	Disagree & totally disagree	19(61.3)	47(39.2)	21(50.0)	6(100.0)	67(43.2)	14(43.8)	40(44.0)	47(46.1)
	P-value	0.073			0.020			0.767	
Currently, there is a possibility of nurse prescribing in critical care and emergency departments.	Agree & totally agree	9(29.0)	71(59.2)	26(61.9)	1(16.7)	87(56.1)	18(56.3)	45(49.5)	61(59.8)
	Disagree & totally disagree	22(71.0)	49(40.8)	16(38.1)	5(83.3)	68(43.9)	14(43.8)	46(50.5)	41(40.2)
	P-value	0.006			0.176			0.149	
I agree with nurse prescribing in critical care and emergency departments.	Agree & totally agree	12(38.7)	65(54.2)	17(40.5)	0(0.0)	80(51.6)	14(43.8)	48(52.7)	46(45.1)
	Disagree & totally disagree	19(61.3)	55(45.8)	25(59.5)	6(100.0)	75(48.4)	18(56.3)	43(47.3)	56(54.9)
	P-value	0.162			0.036			0.286	
There is nothing wrong with prescribing a limited number of emergency medicines after consulting a physician.	Agree & totally agree	27(87.1)	85(70.8)	33(78.6)	0(0.0)	114(73.5)	25(78.1)	70(76.9)	75(73.5)
	Disagree & totally disagree	2(6.5)	13(10.8)	6(14.3)	6(100.0)	17(11.0)	4(12.5)	7(7.7)	14(13.7)
	P-value	0.615			1.00			0.199	
Nurse prescribing limits the physician’s scope of authority.	Agree & totally agree	17(54.8)	60(50.0)	19(45.2)	4(66.7)	77(49.7)	15(46.9)	40(44.0)	56(54.9)
	Disagree & totally disagree	9(29.0)	42(35.0)	12(28.6)	2(33.3)	51(32.9)	10(31.3)	35(38.5)	28(27.5)
	P-value	0.818			1.00			0.086	
Nurse prescribing damages the physician’s position with the patients.	Agree & totally agree	13(41.9)	57(47.5)	21(50.0)	5(83.3)	69(44.5)	17(53.1)	36(39.6)	55(53.9)
	Disagree & totally disagree	9(29.0)	51(42.5)	16(38.1)	1(16.7)	63(40.6)	12(37.5)	40(44.0)	36(35.3)
	P-value	0.825			0.327			0.091	
Nurses do not have the necessary knowledge for prescribing.	Agree & totally agree	23(74.2)	59(49.2)	22(52.4)	5(83.3)	81(52.3)	18(56.3)	48(52.7)	56(54.9)
	Disagree & totally disagree	4(12.9)	46(38.3)	13(31.0)	0(0.0)	54(34.8)	9(28.1)	32(35.2)	31(30.4)
	P-value	0.019			0.206			0.561	
Nurses do not get the necessary preparation to prescribe medicine during their education.	Agree & totally agree	27(87.1)	60(50.0)	23(54.8)	6(100.0)	87(56.1)	17(53.1)	53(58.2)	57(55.9)
	Disagree & totally disagree	1(3.2)	46(38.3)	14(33.3)	0(0.0)	51(32.9)	10(31.3)	27(29.7)	34(33.3)
	P-value	0.001			0.201			0.623	
Nurse prescribing is not in line with the care-oriented nature of the nursing profession.	Agree & totally agree	22(71.0)	67(55.8)	20(47.6)	5(83.3)	86(55.5)	18(56.3)	52(57.1)	57(55.9)
	Disagree & totally disagree	5(16.1)	35(29.2)	15(35.7)	0(0.0)	46(29.7)	9(28.1)	25(27.5)	30(29.4)
	P-value	0.127			0.308			0.785	
Prescribing should only be reserved for nurses with a master’s degree or higher.	Agree & totally agree	15(48.4)	57(47.5)	24(57.1)	3(50.0)	73(47.1)	20(62.5)	43(47.3)	53(52.0)
	Disagree & totally disagree	8(25.8)	23(19.2)	5(11.9)	3(50.0)	30(19.4)	3(9.4)	20(22.0)	16(15.7)
	P-value	0.320			0.118			0.270	

**Note**: ^*^The statistical test used to examine differences between groups was either the Chi-square test or the exact Fisher’s test

^**^The frequency of physicians who selected the option “No opinion” for each statement is not mentioned in the table

The participants’ attitudes revealed several common barriers to the advancement of nurse prescribing in critical care and emergency departments. These included concerns about legal consequences (n = 152, 78.8%), overlapping responsibilities with physicians (n = 143, 74.1%), and a legal vacuum (n = 149, 77.2%) (Table 3).

**Table 3:** Obstacles to the nurse prescribing role from physicians’ viewpoints (N = 193)

Obstacles	Agree & totally agree, n(%)	Disagree and totally disagree, n(%)
Legal consequences	152 (78.8)	23 (11.9)
Legal vacuum	149(77.2)	17 (8.9)
Duties overlap with physicians	143 (74.1)	30 (15.5)
The opposition of physicians	131 (67.9)	37 (30.2)
Contradiction with the caring nature of the nursing profession	130 (62.2)	43 (22.2)
Threatening the patient safety	128 (66.3)	47 (24.3)
Patients’ lack of acceptance	116 (60.1)	38 (19.7)
Insufficient knowledge of disease treatment	111 (57.5)	56 (29.0)
Lack of clinical experience regarding diseases	105 (54.4)	59 (30.6)
Inadequate knowledge of pharmacology	58 (30.0)	117 (60.6)

**Note:** The frequency of physicians who selected the option “No opinion” for each statement is not mentioned in the table

## Discussion

The objective of this study was to examine physicians’ attitudes toward nurse prescribing in critical care and emergency departments. Our findings revealed that a majority of specialist physicians and those with special care fellowships held a positive view regarding the advancement of nurse prescribing in these departments. They expressed agreement with the notion of nurses prescribing a limited number of emergency drugs after consulting with a doctor. However, it is noteworthy that most general practitioners and physicians under the age of 30 were opposed to the development of nurse prescribing. Furthermore, in contrast to the perspectives of specialist and fellowship physicians, over two-thirds of general practitioners disagreed with the idea that nurse prescribing contributes to the professional growth of nursing. Additionally, some physicians under the age of 30 expressed disagreements with the notion that nurse prescribing enhances the quality of patient care. Nevertheless, across other categories such as gender, education, and clinical work experience, more than half of the physicians agreed with this perspective.

Consistent with our findings, a study conducted in Iran in 2022 reported that over two-thirds of critical care physicians and residents expressed trust in the majority of nurses working in intensive care and emergency departments. They also held positive attitudes towards nurse prescribing. More than half of the physicians advocated for critical care nurses to have the ability to prescribe medication. They emphasized that nurse prescribing enhances patient satisfaction and positively impacts the nursing profession [21]. This aligns with the results of Shannon et al.‘s (2011) study, which examined the perspectives of general practitioners and specialist physicians regarding the prescribing role of heart failure specialist nurses. It was found that specialist physicians have closer working relationships with clinical nurses compared to general practitioners, which fosters trust and increases their willingness to accept nurse prescribing [14]. Some opinions expressed by general practitioners in our study are in line with the findings of Mazur et al. (2017) conducted in Poland. Their research explored the attitudes of Polish physicians towards expanding the prescribing capabilities of nurses and midwives. Approximately two-thirds of physicians believed that nurses and midwives should not be granted prescribing licenses and expressed doubt about their readiness and capability. Furthermore, over half of the physicians believed that expanding the prescribing role for nurses has no impact on improving patient care [22]. Similarly, a study by Zarzka et al. (2019) examined the attitudes of Dutch physicians towards the professional development of nurses and midwives. The majority of physicians expressed the belief that nurses and midwives still lack sufficient experience to independently prescribe medication and should only be allowed to prescribe drugs that have already been prescribed by a physician [16]. Based on our research and the studies mentioned, it appears that many physicians support nurses taking on a more advanced role in prescribing medication in specialized and emergency departments. They believe that this could greatly improve patient care. However, there are some physicians who have concerns about the ability of nurses to prescribe effectively. To address these concerns, it may be beneficial to enhance the qualifications of nurses working in these settings and grant them the necessary authority to prescribe medication.

In our study, a significant proportion of physicians across various educational levels expressed the belief that nurse prescribing is incongruent with the care-oriented nature of the nursing profession. However, a study conducted by Mazur et al. (2017) in Poland reported that the majority of respondents agreed with this perspective. They acknowledged that granting new powers, such as prescribing, enhances the professional credibility of nurses and midwives. Additionally, approximately half of the respondents believed that nurse and midwife prescribing reduces the burden of responsibility on physicians [22]. Haririan et al.‘s study in Iran (year not provided) revealed that the majority of specialized care physicians perceived that nurse prescribing of medications contributes to increased patient satisfaction and positively impacts the nursing profession [21]. Similarly, a study conducted in the Netherlands in 2014 demonstrated a positive outlook from both physicians and nurses regarding nurse prescribing [6]. In an English qualitative study from 2020, physicians expressed support for the role of nurse prescribing [14]. Another qualitative study from 2009 indicated that all English physicians and nurses emphasized the necessity and significance of nurse prescribing [23]. However, the same study highlighted physicians’ concerns about trust in nurses, the selection of nurses for teaching drug administration principles, and the impact of nurse prescribing on physicians’ responsibilities [23]. Overall, while physicians generally agree with nurse prescribing, they have valid concerns that should be taken into account by senior nursing managers.

In the present study, a majority of physicians acknowledged that nurses in critical care and emergency departments have a history of prescribing medication without consulting them, leading them to consider the possibility of delegating this responsibility to nurses in these departments. Interestingly, even in countries where nursing prescribing is not legally permitted, nurses in intensive care units are compelled to prescribe medication in emergency situations to save patients’ lives [9, 24]. As a result, it becomes imperative to ensure that critical care and emergency nurses possess the necessary qualifications for prescribing medication. In this regard, it is crucial to precisely determine the scientific qualifications required for nurses who seek to obtain a prescription license [25]. The development of the prescribing role for critical care and emergency nurses with a master’s degree, extensive clinical experience, and professional qualifications is absolutely essential. Such development holds the potential to benefit patients in critical care and emergency departments by improving their access to timely and appropriate medication.

In our study, a majority of physicians expressed the belief that nurses holding a master’s degree or higher should be responsible for prescribing medication. It is widely recognized that high academic qualifications and substantial clinical experience are crucial for ensuring safe and effective prescription practices [26]. Supporting this notion, a qualitative study conducted in Ireland in 2019 highlighted the importance of including a prescription course within the post-graduate curriculum of emergency and critical care nursing disciplines. Such a course would equip nurses with the necessary qualifications to engage in prescribing medication [27]. In line with this perspective, recent changes in the nursing postgraduate curriculum in Iran demonstrate a commitment to specialization within the nursing field and the advancement of roles, including prescribing. These developments hold the potential to elevate the nursing profession and its impact on healthcare delivery.

The findings of the current study highlight several benefits of nurse prescribing from the perspective of physicians. These benefits include the advancement of the nursing field, improvement in the quality of care services, increased professional independence, and the potential to save patients’ lives in emergency situations. These results align with a study conducted in the Netherlands in 2014, where both physicians and nurses acknowledged that nurse prescribing promotes nurses’ independence [6]. Similarly, English physicians in a 2011 study emphasized the numerous advantages of nurse prescribing for patients, including increased patient satisfaction and a reduced workload for physicians. By reducing direct patient contact, nurse prescribing also saves valuable time for general practitioners and specialists. While general practitioners were primarily interested in the potential workload reduction, specialist physicians recognized broader positive consequences for patients resulting from the expansion of the prescribing role for nurses [14]. In an Irish study from 2008, nurses expressed their belief that nurse prescribing enhances the quality of healthcare, utilizes professional skills, and increases nursing autonomy [28]. Another study in Ireland conducted in 2018 further supported the positive effect of nurse prescribing on nursing professional development. The development of the prescribing role for nurses not only plays a crucial role in attracting and retaining the nursing workforce, but it also facilitates access to essential medical services and ensures the delivery of safe and effective care [28]. Nurse prescribing has significant advantages, including improving healthcare quality, professional autonomy, and patient outcomes.

In our study, physicians identified several common barriers to nurse prescribing, including concerns about legal consequences, interference with physicians’ duties, and a legal vacuum. Nurse prescribing entails a significant increase in legal and professional responsibilities [16]. A study conducted in Ireland in 2008 highlighted the lack of legality as an obstacle to nurse prescribing, emphasizing the importance of legalizing nursing prescriptions to alleviate concerns about legal consequences [29]. Physicians’ resistance also emerges as a significant barrier to the development of the nurse-prescribing role [7, 12, 16]. Given that prescribing is considered one of the primary responsibilities of physicians [11], this resistance is to be expected. Polish physicians in a study from 2019 expressed the belief that nurses are still inadequately prepared to prescribe medication, with only a few capable of re-prescribing physicians’ prescriptions [16]. However, in contrast to this study, Naderi et al.‘s research in 2021 found that over two-thirds of nurses reported having the necessary qualifications to prescribe medication [26]. Concerns about the quality of care and patient safety were the most common among physicians in England regarding nurse prescribing, as revealed in a 2006 study [6]. Similar concerns about the safety of nurse prescribing were expressed in two other studies conducted in England [30, 31]. The development of the nurse prescribing role faces numerous challenges, necessitating nursing policymakers to address legal barriers and seek the agreement of medical policymakers regarding this role. Nurses should also strive to acquire the requisite scientific qualifications to fulfill this crucial role.

### Limitations

This cross-sectional study had certain limitations that should be acknowledged. Firstly, the study design prevented establishing a cause-and-effect relationship between the variables under investigation. Secondly, the data collection method relied on self-administered questionnaires, which made it challenging to verify the accuracy of the responses. However, steps were taken to mitigate this limitation by clearly communicating the research objectives to physicians and reassuring them about the anonymity of their questionnaire submissions.

## Conclusions

In the present study, a majority of physicians across various age groups, genders, educational backgrounds, and clinical work experience levels expressed agreement with nurse prescribing of drugs in emergency situations, recognizing its importance for saving patients’ lives. Notably, physicians with specialized education and fellowships in specialized care demonstrated a higher inclination towards expanding the role of nurse prescribing in special care and emergency departments. Conversely, a significant proportion of general practitioners and physicians under the age of 30 exhibited opposition towards the advancement of nurse prescribing responsibilities. Legal consequences, legal gaps, and interference with physicians’ duties were identified as the primary obstacles to the development of the nurse-prescribing role from the physicians’ perspective. To facilitate the development of the nurse-prescribing role in critical care and emergency departments, it is essential for nurses to acquire the necessary scientific qualifications. Nursing education policymakers should therefore incorporate relevant course units on nurse prescribing into the curriculum of various nursing programs, especially at the master’s level in critical care and emergency nursing. Nursing managers should also prioritize postgraduate programs and clinical qualifications for nurses holding a master’s degree. Additionally, nursing organizations should actively work towards removing legal barriers that impede the nurse-prescribing role. It is recommended that similar studies be conducted in other regions to further explore and address the challenges associated with nurse prescribing. Furthermore, the professional competence of nurses in critical care and emergency departments, specifically in the management of common diseases and pharmacology, should be periodically evaluated through standardized and national examinations.

## Data Availability

The datasets analyzed in the current study are available upon reasonable request from the corresponding author.
